# Ureteroscopic Management of Chronic Unilateral Hematuria: A Single-Center Experience over 22 Years

**DOI:** 10.1371/journal.pone.0036729

**Published:** 2012-06-08

**Authors:** Motoo Araki, Shinya Uehara, Katsumi Sasaki, Koichi Monden, Masaya Tsugawa, Toyohiko Watanabe, Manoji Monga, Yasutomo Nasu, Hiromi Kumon

**Affiliations:** 1 Department of Urology, Okayama University Graduate School of Medicine, Dentistry and Pharmaceutical Sciences, Okayama, Japan; 2 Glickman Urological and Kidney Institute, Cleveland Clinic, Cleveland, Ohio, United States of America; Harvard Medical School, United States of America

## Abstract

**Objective:**

To analyze the short and long term safety and efficacy of ureteroscopic evaluation and management of chronic unilateral hematuria.

**Methods:**

We retrospectively reviewed patients with chronic unilateral hematuria from 1987 to 2008. The distal to middle ureter was evaluated with a semi-rigid ureteroscope without a guidewire. Subsequently, the flexible ureteroscope was advanced into the upper ureter to the renal pelvis using a low-pressure automated irrigant system (Uromat™). Lesions identified ureteroscopically were treated with diathermy fulguration.

**Results:**

One hundred and four (56 male, 48 female) patients were identified, with a median age of 37 (14–80) years and median follow-up of 139 (34–277) months. The median preoperative duration of gross hematuria was 5 (1–144) months. Endoscopic findings included 61 (56%) minute venous rupture (MVR; a venous bleeding without clear abnormalities), 21 (20%) hemangioma (vascular tumor-like structure), 3 (3%) varix (tortuous vein), 1 (1%) calculus and 18 (17%) no lesions. The incidence of “no lesions” was less in the recent 12 years (9%) than the first 10 years (27%), while the incidence of MVR increased from 40 to 66% (p<0.05). All patients were treated endoscopically. Immediate success rate was 96% (100% in the recent 12 years). Long-term recurrent gross hematuria rate was 7%. Six resolved spontaneously and only 1 required ureteroscopy, revealing a different bleeding site.

**Conclusion:**

Ureteroscopy and diathermy fulguration is highly useful for evaluation and treatment of chronic unilateral hematuria. Sophisticated technique and improved instrumentation contributes to a better outcome.

## Introduction

Chronic unilateral hematuria poses a diagnostic and therapeutic dilemma. [1], [2], [3] It is characterized by intermittent or continuous gross hematuria that cannot be diagnosed with standard radiologic and hematologic tests. [4] Cystoscopy can reveal that hematuria is renal in origin, and may demonstrate lateralization to one or both ureteral orifices. Chronic unilateral hematuria is also known as unilateral essential hematuria. [1] Historically, some patients have underwent nephrectomy for hemostasis or possible malignancy. [5]

To avoid unnecessary nephrectomy, several investigators have reported the utility of ureteroscopy for evaluating and treating this disease. [1], [2], [3], [4], [6], [7] Initially, there were some limitations such as size of instrumentation, clarity of vision and modality of hemostasis. Literature reviews reported successful outcomes were between 58 and 87%. [8]

To achieve higher successful treatment rates, we have been refining our instrumentation and technique. Over the years, we have learned that the small tips are very important treating this entity including when and how to use guidewire, the control of irrigation pressure, the order of ureteroscopic observation and hemostasis modality.

We report our technique and results over a 22-year experience.

## Methods

### Preoperative Diagnostic Work-up

Between 1987 and 2008, 104 patients with chronic unilateral hematuria were referred to our hospital for ureteroscopic evaluation. Radiology and hematology tests were normal and failed to reveal the source of hematuria. Cystoscopy revealed the hematuria from 1 side in all patients except 1 patient who had bilateral bleeding. Evaluation included cystoscopy, intravenous pyelogram (IVP), ultrasonography, computerized tomography (CT). Angiography was performed in cases when arteriovenous malformation (AVM) or arteriovenous fistula (AVF) was suspected on CT scan (shunt) or ultrasound (mosaic pattern). Hematology tests included prothrombin, partial thromboplastin and bleeding times and complete blood count. Series of urine cytology and urine culture were also negative. Exclusion criteria included urinary tract infection documented by urine analysis and urine culture (n = 1). AVM and AVM that were diagnosed by angiography were excluded as these were treated by embolization by interventional radiologist (n = 1).

### Technique

Following the prophylactic cefazolin administration, the procedure is initiated with cystoscopy under general anesthesia. Our technique has been evolved over 22 years, and is summarized in Table 1. Specifically, as smaller endoscopic instrumentation became available, the need for dilation of the ureteral orifice and/or peel-away sheath was eliminated. Our irrigation system changed from static to dynamic pressure controlled system, Uromat™ (Karl Storz, Tuttlingen, Germany).

**Table 1:** The changes of ureteroscopic techniques.

	I			II
	I-a	I-b	I-c	
	(July 1987∼)	(April 1991∼)	(March 1994∼)	(November 1997∼)
Dilation of ureteral orifice	(+)	(+)	(−)	(−)
Peel-away sheath	(+)	(−)	(−)	(−)
Endoscope	URF	AUR-9	Multi-scope	Multi-scope
(Diameter,	(13.2, 5.1 Fr)	AUR-8	(7/12, 4.0 Fr)	(6/7.4, 4.0 Fr)
working channel)	AUR-9	(8.5, 2.5 Fr)	↓	↓
	(9.8, 3.6 Fr)		AUR-9,8	AUR-7
				(7.2, 3.6 Fr)
				URF-P3
				(6.9, 3.6 Fr)
Control of the pressure of theirrigating fluid	Static	Dynamic (Uromat™)	Dynamic (Uromat™)	Dynamic (Uromat™)

Since November 1997 (II period), our technique has been fixed. Following cystoscopy, the distal to middle portion of the ureter (L4–5 level) is inspected using a 6.9 Fr semi-rigid ureteroscope without a guidewire. Semi-rigid ureteroscope also gently dilates the ureteral orifice and helps the following flexible ureteroscope insertion. Subsequently, an initial guidewire is placed under the fluoroscopic guidewire to the level of endoscopic inspection. A special caution should be taken not to advance a guidewire beyond the middle ureter (L4–5 level) in order to avoid an inadvertent injury to the collecting system. Semi-rigid ureteroscope is removed and flexible ureteroscope ((AUR-7, (size: 7.2 Fr, working channel: 3.6 Fr) or URF-P3 (6.9 Fr, 3.6 Fr)) is passed over the guidewire into the ureter under fluoroscopic guidance, again with a caution not to advance a guidewire beyond the middle ureter. The guidewire is then removed. The proximal ureter and collecting system are inspected with flexible ureteroscope. Systemic evaluation of entire collecting system should be performed in order: upper, middle, and lastly lower to avoid an inadvertent urothelial bleeding. If the flexible ureteroscope is passed initially into the lower pole, the shaft of the flexible ureteroscope can cause bleeding from the upper pole infundibulum and can mislead the examiner. A biopsy is performed with a 3 Fr cup forceps though the ureteroscope if malignancy is suspected. Continuous irrigation is maintained through the working channel of the ureteroscope in a lowest flow pressure (40 mm Hg) with Uromat™ (Karl Storz). Irrigant containing iodinated contrast material (17%) is used to outline the collecting system for radiographic monitoring. When a source of bleeding it not clear, Uromat is discontinued and ureteroscopy is performed without irrigation.

Once lesions are identified, they are cauterized by 2 or 3 Fr (30 W) Bugbee electrode through the working channel of flexible ureteroscope. At the end of procedure, a single-J stent (6 Fr in diameter, external stent) is placed for a several days only in cases which required ureteral dilation. Follow-up includes urinalysis at 4 weeks and every 3 months in the first year and every year post-surgery. Renal ultrasound was performed every clinical visit. In cases of malignancy, urine cytology was obtained every 3 months and ureteroscopy was performed every 3 months in the first 2 years, every 6 months in 3 to 5 years post-surgery and every year thereafter. All patients completed a minimum follow-up of two years.

Okayama University Hospital institutional review board approval was obtained for this study. Patients are consented at admission before surgery with written documents for study.

The statistical analysis was performed by StatView software. P values were derived from the chi-square test or Fisher’s exact test (categorical variables) or Student’s t test (continuous variables). All statistical tests were two-sided.

## Results

One hundred and four patients were identified, with a median age of 37 (range 14–80) years (Table 2). Median follow-up was 139 (34–277) months. Patients had gross hematuria prior to surgery for 5 (range 1–144) months. Sixty-eseven (64%) patients had bleeding in the left, 37 (36%) in the right and 1 (1%) in both sides. Prior to ureteroscopy at our institution, 39 (38%) of patients had received hemostatic medication (carbazochrome sodium sulfonate and/or amstat) either orally or intravenously. 17 (16%) of patients had underwent the instillation of silver nitrate into the renal pelvis at outside hospitals. One patient (1%) had undergone ureteroscopy with an unsuccessful outcome at an outside hospital. The proportion of patients treated with silver nitrate prior to referral to our center has decreased from 33 to 6% over the years (p<0.005, Table 3).

**Table 2:** Patient characteristics.

No. patients	104
No. men/women	56/48
Median age (range)	37 (14–80)
No. left/right/bilateral	67/37/1
Median duration of hematuria (range) (mos)	5 (1–144)
Median follow-up (range) (mos)	139 (34–277)

**Table 3:** Treatment prior to ureteroscopy at outside hospital.

N (%)	Hemostatic medicine	Silver nitrate	Ureteroscopy	None	Total
Period I	15 (38)	13 (33)	0	12 (30)	40 (100)
Period II	24 (36)	4 (6)	1 (1)	38 (55)	64 (100)
Total (N = 104)	39 (38)	17 (16)	1 (1)	47 (45)	109 (100)
p-value (I vs II)	1	<0.001	1	<0.05	N/A

**Figure 1:**
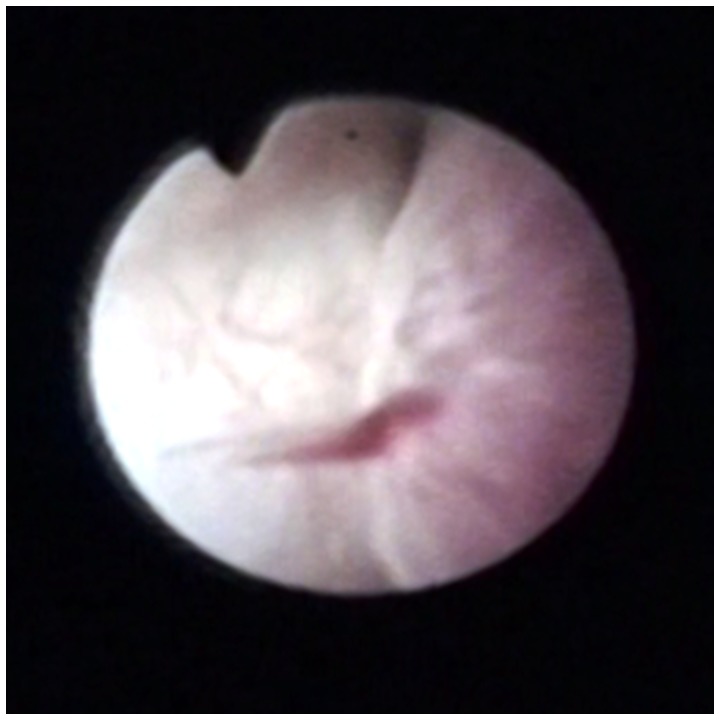
Major endoscopic findings. Minute venous rupture (MVR: a bleeding without clear abnormalities).

The entire collecting system was completely inspected in all patients. The lesions identified included 61 (56%) minute venous rupture (a small venous bleeding without clear abnormalities, MVR), 21 (20%) hemangioma (vascular tumor-like structure), 3 (3%) varix (tortuous vein), 1 (1%) calculus. The incidence of “no lesions” was less in the recent 12 years or II period (9%) than the first 10 years or I period (29%, p<0.05), while the incidence of MVR increased from 40 to 66% (p<0.05). All patients were treated successfully endoscopically (Table 4). The major endoscopic findings are demonstrated in Figure 1.

**Table 4:** Endoscopic findings in patients with chronic unilateral hematuria.

N (%)	Minute venous rupture	Hemangioma	Varix	Calculus	No lesion
Period I(N = 40)	16 (40)	10 (25)	1 (3)	1 (2)	12 (29)
Period II (N = 64)	45 (66)	11 (16)	2 (3)	0	6 (9)
Total (N = 104)	61 (56)	21 (20)	3 (3)	1 (1)	18 (17)
p-value (I vs II)	<0.005	0.47	1	0.38	<0.05

**Figure 2:**
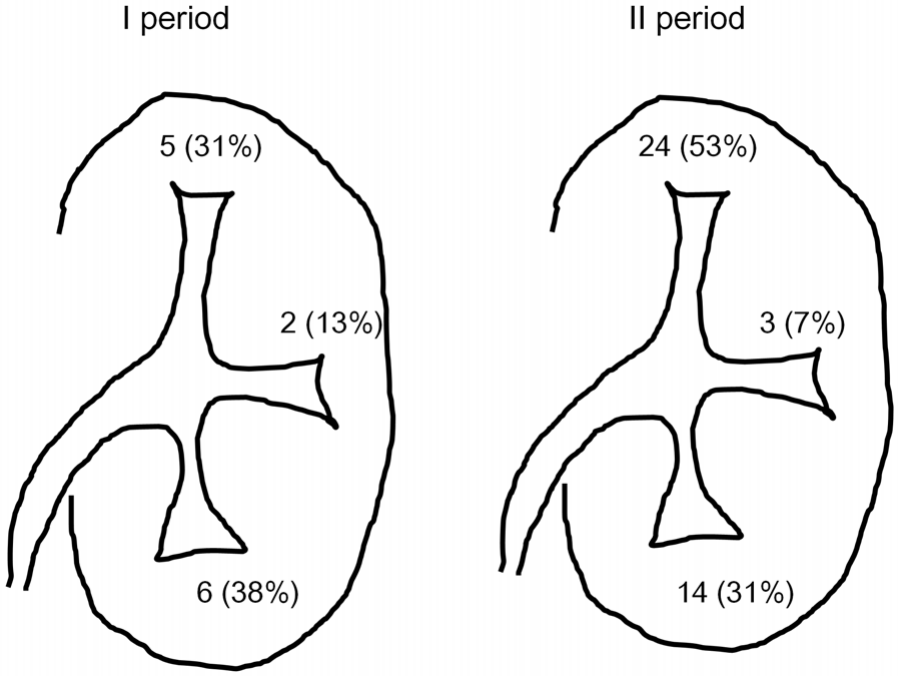
Locations of minute venous rupture (MVR). I period: Multiple lesions: 1 (6%) (Rt. upper pole and lower pole calices). Not documented:2 (13%). II period: Multiple lesions: 3 (7%) Case 1 (rt. upper pole and middle pole calices), Case 2 (rt. Upper pole and lower pole calices), Case 3 (a bilateral case: rt. upper pole, lt. upper and lower pole calices). Not documented:1 (2%).

The location of MVR were documented in 59/62 (95%) cases (Figure 2). The site of MVR located primarily in upper pole calices (29 (48%)), followed by lower pole calices (20 (33%)) and middle pole calyces (5 (8%)). Bleeding from multiple sites was identified in 3 unilateral cases and 1 bilateral case. The location of MVR did not differ significantly between I and II periods.

**Table 5:** Recurrent gross hematuria and adverse events.

N (%)	Immediate treatment failure	Incidence of recurrent gross hematuria	Outcome of recurrent gross hematuria		Adverse events	
			Spontaneous resolution	URS	Extravasation	Pyelonephritis
I (N = 40)	4 (10)*	5 (12)	4 (10)**	1 (2)***	9 (22)	4 (10)
II (N = 64)	0	2 (3)	2 (3)#	0	2 (3)	0
p-value (I vs. II)	<0.05	0.1	0.2	0.38	<0.05	<0.05

* One required second ureteroscopy. Other 3 underwent successful percutaneous nephroscopic electrocautery.

** Recurrent gross hematuria occurred at 1 month, 1, 2 and 2 year post-surgery, respectively. All resolved spontaneously.

*** One had a bleeding from a different site at 2 years post-surgery. Therefore this does not seem to be a treatment failure.

# Recurrent gross hematuria occurred in 8 and 10 year post-surgery, respectively. Both resolved spontaneously.

Immediate success rate, defined as absence of gross hematuria within a month post-surgery, was 100/104 (96%). Four required another surgery within a month, one underwent a second ureteroscopy and three underwent percutaneous nephroscopic electrocautery because they were too large to treat ureteroscopically (All three were in I-period). No immediate treatment failure was identified in the recent 12 years.

7/104 (7%) experienced recurrent gross hematuria, defined as a bleeding more than one month after successful initial treatment. The incidence of recurrent gross hematuria has decreased to 3% (2/68) from 12% (5/40), although it is not significant (p = 0.1). Only 1 required ureteroscopy for hemostasis at 2 year post-surgery, revealing a different bleeding site (right upper and lower pole) from the original (right middle pole). Other 6 cases stopped bleeding spontaneously without ureteroscopy.

Adverse events include 11 (10%) extravasations and 4 (4%) pyelonephritis (Table 5). All were treated conservatively: patients noted to have extravasation were treated with ureteral stent and pyelonephritis was treated with antibiotics. The incidence of those adverse events decreased over the two study periods (p<0.05).

## Discussion

Chronic unilateral hematuria is also known as unilateral essential hematuria, [5] lateralizing essential hematuria, [6], [9] and benign lateralizing hematuria. [10]

More than 200 cases of chronic unilateral hematuria have been reported to date [8], [11] but there has been only a few reports in the last 5 years. [2], [12], [13] This report provides the history of the diagnosis and treatment of chronic unilateral hematuria. Our series has the longest-follow-up with the largest series of patients from a single institution. Lano et al. reported 54 patients with chronic unilateral hematuria in 1979. [5] Since ureteroscopy was not yet available that time, ancillary studies such as renal arteriography, renal biopsy, and repeated excretory urography and cystoscopic examinations were performed, but were not helpful in establishing a cause. However, the advent of endoscopy has changed the approach to diagnosis of chronic unilateral hematuria. The etiology has been revealed to include not only hemangioma but minute venous rupture, varix or others. We have been working on this entity since 1987 and published our initial report in 1990. Flexible endoscopy has also revolutionized the treatment of chronic unilateral hematuria. The traditional management of recurrent, prolonged bleeding has been bed-rest, blood transfusion, the instillation of silver nitrate into the renal pelvis. The instillation of silver nitrate into the renal pelvis had been the most common conservative management of chronic unilateral hematuria, first described by Diamond. Ten mL of silver nitrate (0.25 to 1%) was instilled into the upper collecting system in the original articles. In our series, 17 (16%) of patients underwent the instillation of silver nitrate into the renal pelvis at outside hospitals prior to ureteroscopy. Silver nitrate is often associated with a number of serious side effects, including sepsis, interstitial nephritis, anuria, pyonephrosis, and ureteral strictures. [14] Due to our continuous warning of these side effects in our society, the number of silver nitrate treatment decreased from 34 to 6% over the years in our series. It should be contraindicated for the treatment of chronic unilateral hematuria. More aggressive measures have included total and partial nephrectomy. In contrast, Lano et al. reported that only 56% continued to have intermittent or persistent hematuria with conservative observation. [5] In our series, 6/7 (86%) patients with recurrent gross hematuria had spontaneous resolution. It seems that partial/total nephrectomy should be the avoided.

The recent introduction of flexible ureteroscopy has allowed successful nephron-sparing endoscopic treatment with minimum complications. The major endoscopic finding was MVR (56%) in our series. MVR appears as a bleeding without clear abnormalities typically from the tip of the papilla. In contrast, hemangiomas appear as either small red or bluish spots at the tip or base of a papilla. [3] Our results echoes those reported in other reports. [15], [16] However, there are a few reports where hemangioma was the major endoscopic findings. [3], [10] The second most common endoscopic finding was hemangioma (21%) in our series. The different results may be related to the genetic or ethnic make-up of our study populations. There were no major (sepsis, blood transfusions, death) complications in our series. Uromat™ plays an important role by controlling the pressure of irrigation fluid the lowest level (40 mm Hg) to prevent pyelonephritis and extravasation.

There are several pearls for evaluation and treatment of chronic unilateral hematuria.

1. The meticulous endoscopic technique: Examination should be performed in order: upper, middle, and lastly lower because if the flexible ureteroscope is passed first into the lower pole, the shaft of the endoscope can bruise the urothelium. Prior to flexible ureteroscopy, inadvertent injury should be avoided. In our recent technique, the guidewire is never advanced into the collecting system beyond the point of endoscopic inspection. After semi-rigid ureteroscopy at the distal and middle ureter, the guidewire is kept at the site where semi-rigid ureteroscopy has been completed under fluoroscopy. Guidewire can be easily lost in this technique so that special caution is required. We also have to keep in mind that the guidewire can go up easily to the collecting system. If that happens, we should regard MVR in the upper pole as an inadvertent injury.
2. The control of irrigation pressure: Minute venous bleeding is such a delicate lesion. The increased intraluminal pressure during ureteroscopy can easily seal a small venous rupture. Literature review revealed that no lesion was seen in 16% of cases of chronic unilateral hematuria. Even in the most recent report of chronic unilateral hematuria, there were 5 (21%) patients with no lesions at the time of ureteroscopy. All 5 had no recurrent bleeding post-surgery. [2] Other series reported similar results. [2], [10] The venous-caliceal communication may have been closed by tamponade effect of the increased intraluminal pressure. However, for a diagnostic purpose, gentle irrigation is crucial in this technique. In most reports, irrigation is maintained by gravity and manual syringe injection. [2], [5], [6] In our recent series, maintenance of a minimum irrigation pressure is maintained at the lowest (40 mm Hg) with Uromat™ throughout the procedure. It is suggested that the utilization of this device have contributed to a decreased incidence of “no lesion” and increased incidence of MVR. We also create a negative pressure with syringe aspiration through the working channel when bleeding is unclear. With those techniques, our rate of “no lesion” has decreased over time from 29% to 9%.
3. The improvement of endoscopic instruments: Endoscope has been miniaturized. The size of semi-rigid ureteroscope has changed from 13.2 Fr to 6 Fr and that of flexible ureteroscope has changed from 9.8 Fr to 6.9 Fr. Illumination has also improved to obtain a better endoscopic vision. New generation flexible ureteroscope with two deflectable segments allows complete examination of the collecting system, especially of the lower calyx. Digital ureteroscope is a major advance in endourology and has superior visualization compared with a conventional fiber ureteroscope. We believe that it will help the diagnosis and the treatment of chronic unilateral hematuria.
4. Semi-rigid ureteroscopy prior to flexible ureteroscopy: Although flexible ureteroscope is miniaturized, the flexible ureteroscope will not pass the ureteral orifice without dilation in most of the Japanese population. Therefore, we routinely utilize semi-rigid ureteroscope prior to flexible ureteroscope. The elimination of ureteral dilation is important in the diagnosis of chronic unilateral hematuria because dilation of the ureter prior to ureteroscopy creates iatrogenic change in the ureter. The use of semi-rigid ureteroscope achieves both diagnosistic ureteroscopy and gentle dilation of the ureter.
5. A patient preparation: It is extremely difficult to evaluate bleeding without gross hematuria at the time of ureteroscopy. A patient should be very active prior to surgery if he does not have an ongoing gross hematuria. For example, a patient is asked to climb a few flights of stairs prior to ureteroscopy.

We utilize 2 or 3 Fr electrode for hemostasis. Diathermy fulguration has been used in more than 80% of reported endoscopic treatments. [8] It is safe, inexpensive, widely available and already established in the treatment of bladder lesions. Holmium:YAG or Nd: YAG laser treatment has been also reported. [2], [3], [6], [13] Literature review revealed successful outcome was between 58 and 87% but did not discuss the superiority of laser vs. electrocautery treatment modality. [8] However, successful rates of diathermy fulguration is as high as 90% in our series (100% in the last 12 years). In addition, the expense of diathermy fulguration is much less in Japan than Holmium:YAG or Nd: YAG laser ablation is the cost. [8] With those reasons, we favor diathermy fulguration over Holmium:YAG or Nd: YAG laser ablation for the treatment of chronic unilateral hematuria.

The pathogenesis of MVR or hemangioma is poorly understood. Venous hypertension, renal pelvic inflammation, infection, thrombosis, trauma from calculi and secondary arterial renal venous hypertension are all possible explanations.^8,19^ Further investigation of calyceal urodynamics or renal venous hemodynamic may be necessary.

In conclusion, chronic unilateral hematuria has been a difficult problem of diagnosis and treatment with minimum complications. Ureteroscopy is highly useful for evaluation and treatment of chronic unilateral hematuria. In our series, the major endoscopic finding was minute venous rupture (56%). The advent of miniaturized ureteroscopes with technical improvement and experience has contributed to a more accurate etiology and treatment of chronic unilateral hematuria. Diathermy fulguration is inexpensive and effective treatment modality for the treatment of this entity.
